# Naturalistic study of guideline implementation tool use via evaluation of website access and physician survey

**DOI:** 10.1186/s12911-016-0404-2

**Published:** 2017-01-13

**Authors:** Melissa J. Armstrong, Gary S. Gronseth, Richard Dubinsky, Sonja Potrebic, Rebecca Penfold Murray, Thomas S. D. Getchius, Carol Rheaume, Anna R Gagliardi

**Affiliations:** 1 Department of Neurology, University of Florida College of Medicine, Gainesville, FL USA; 2 University of Maryland School of Medicine, Baltimore, MD USA; 3 Department of Neurology, University of Kansas Medical Center, Kansas City, KS USA; 4 Department of Neurology, Kaiser Permanente - Los Angeles Medical Center, Los Angeles, CA USA; 5 American Academy of Neurology, Minneapolis, MN USA; 6 Toronto General Research Institute, University Health Network, Toronto, Canada

**Keywords:** Practice guidelines, Guideline implementation, Information dissemination, Internet, Surveys and questionnaires

## Abstract

**Background:**

Clinical guidelines support decision-making at the point-of-care but the onus is often on individual users such as physicians to implement them. Research shows that the inclusion of implementation tools in or with guidelines (GItools) is associated with guideline use. However, there is little research on which GItools best support implementation by individual physicians. The purpose of this study was to investigate naturalistic access and use of GItools produced by the American Academy of Neurology (AAN) to inform future tool development.

**Methods:**

Website accesses over six months were summarized for eight AAN guidelines and associated GItools published between July 2012 and August 2013. Academy members were surveyed about use of tools accompanying the sport concussion guideline. Data were analyzed using summary statistics and the Chi-square test.

**Results:**

The clinician summary was accessed more frequently (29.0%, *p* < 0.001) compared with the slide presentation (26.8%), patient summary (23.2%) or case study (20.9%), although this varied by guideline topic. For the sport concussion guideline, which was accompanied by a greater variety of GItools, the mobile phone quick reference check application was most frequently accessed, followed by the clinician summary, patient summary, and slide presentation. For the sports concussion guideline survey, most respondents (response rate 21.8%, 168/797) were aware of the guideline (88.1%) and had read the guideline (78.6%). For GItool use, respondents indicated reading the reference card (51.2%), clinician summary (45.2%), patient summary (28.0%), mobile phone application (26.2%), and coach/athletic trainer summary (20.2%). Patterns of sports concussion GItool use were similar between respondents who said they had and had not yet implemented the guideline.

**Conclusions:**

Developers faced with resource limitations may wish to prioritize the development of printable or mobile application clinician summaries, which were accessed significantly more than other types of GItools. Further research is needed to understand how to optimize the design of such GItools.

## Background

Guidelines synthesize research evidence to inform decision making by health care policy-makers, managers and providers, and they are produced in ever-increasing numbers by government, non-profit and professional organizations [1]. Compliance with guidelines is variable and often poor, thus limiting the benefits of evidence-based care on patient safety and outcomes [2–4]. There are many potential and often co-existing reasons for poor guideline compliance including the characteristics of guidelines, patients and providers, and other health system factors that influence resources and costs [5, 6]. Furthermore, many guidelines are not actively implemented because developers often have few dedicated resources to support implementation efforts [7–9]. Repeat surveys of Canadian guideline developers in 1994 and 2005 found that guideline implementation had decreased [8]. A survey of international guideline developers revealed that, given their lack of resources, they expected users to assume the responsibility for implementing guidelines [9].

Thus the onus is on target users to implement guidelines. However, focus groups found that health professionals were frustrated and uncertain about how to implement guidelines [10]. A systematic review of studies that evaluated guideline implementation found that, even when awareness of and agreement with guidelines were high, adoption and adherence were comparatively lower [11]. Hence, users require support for guideline implementation. There is empirical evidence that the inclusion of implementation instructions or tools in or with guidelines is associated with guideline use. For example, a systematic review of 68 studies of provider adherence to asthma guidelines found that decision support tools (electronic or paper-based guideline summaries, algorithms, history-taking template, asthma status reminders) increased prescribing and provision of patient self-education or action plans, and was the only intervention studied that reduced emergency department visits [12]. A Cochrane systematic review of eight studies found that print summaries improved compliance with care delivery recommendations [13]. As a result, experts have advocated for developers to provide users with guideline implementation tools (GItools) such as summaries, checklists, algorithms, or decision-making aids for patients or providers [14, 15].

Research shows that few guidelines provide users with such GItools. Guidelines published in 2008 or later were high in quality for scope and purpose, stakeholder involvement, rigor of development and clarity of presentation, but were consistently lacking in applicability, which refers to implementation instructions or tools, and their applicability had not improved compared with guidelines published in 2007 or earlier [16]. Interviews with 30 guideline developers or implementers from government and professional societies in seven countries revealed that few had developed GItools [7]. However, they described a demand for GItools among target users of their guidelines and requested guidance for developing GItools. Analysis of guideline development manuals found they were lacking in instructions for generating GItools [17].

Recent work with international guideline developers has identified ideal characteristics of GItools [18] and processes and practical considerations for developing GItools [19]. Although research has associated GItools with guideline use [12, 13] and resources are now available to help guideline developers create and package GItools with their guidelines [18, 19], there remains a need to ensure that GItools are relevant and useful to health professionals. There are many types of GItools that can potentially be used in different ways to achieve various outcomes. For example, a guideline summary might be used by an individual physician at the point of care as a reminder of the key recommendations; a patient summary might be used by an individual physician at the point of care to engage patients in informed or shared decision making; educational resources might be used by an individual physician for self-directed learning, or by teams as the basis for training, continuing professional development, or quality improvement planning; and checklists, algorithms or performance measures might be used by a quality improvement team to integrate guideline recommendations with clinical decision support systems.

Research to date has examined the use of specific types of implementation tools that were under evaluation in the context of investigations [12, 13]. In one systematic review, investigators evaluated adherence to asthma guidelines as measured by healthcare process outcomes. The review found that clinical pharmacy support, decision support tools, and feedback and audit strategies were the strategies most likely to improve adherence in the context of research studies (*n* = 68, half randomized controlled trials, half pre-post studies) [12]. A Cochrane review of interventions to improve systematic review use in healthcare decision-making identified only 8 studies investigating the effectiveness of implementation interventions for systematic reviews. Systematic review physician summaries (print bulletins) resulted in greater adherence to evidence-based practice, though other specific contextual factors (e.g., media coverage, funding changes) may also have played a role [13]. Little research has examined naturalistic access of GItools, which might provide insight on how they are used in practice. Given that one barrier to implementation activities for guidelines is lack of funding [7], understanding real-life access of GItools can help developers identify what GItools may be most important to end-users.

The purpose of this study was to explore the types of GItools that were most accessed in the six months following guideline publication. In this context, GItool access is assumed to imply use, which could reflect either instrumental use, where the tool is used for decision-making with a patient, or conceptual use, where the tool is used to influence the user’s thinking without immediate application [20]. This information could be used by guideline developers with limited funding to help prioritize the types of GItools they develop, and focus their efforts to optimize the content, format and delivery of the specific types of GItools that are relevant and useful to physicians. This information could also be used by researchers to identify relevant theories and interventions that can be used in future research to more rigorously evaluate the implementation and impact of specific types of GItools.

## Methods

### Approach

The American Academy of Neurology (AAN) has developed guidelines since the mid-1990s and has produced 70 guidelines since 2004. To supplement a variety of dissemination and implementation strategies, most AAN guidelines offer GItools including a clinician summary, patient/family summary, a clinical case example and an educational slide set. Select guidelines may also include additional GItools such as algorithms, checklists, and/or mobile phone applications. All guidelines and accompanying GItools are freely available on the AAN website [21]. We analyzed AAN website usage statistics to identify the most frequently accessed GItools, and analyzed responses about GItools from an AAN membership survey that was undertaken to plan future guideline dissemination strategies. Ethical approval to conduct this study was provided by the Institutional Review Board of the University of Maryland School of Medicine.

### GItool accesses

All AAN guidelines published between July 2012 and August 2013 were included and, for each of these, accesses to the guideline and its GItools in the six calendar months following publication were noted [22–29]. A six-month window was chosen as this was felt to reliably represent the initial uptake of GItools in response to guideline publication and dissemination strategies including the initial press release, membership emails with key messaging, and podcasts linked to the journal (Neurology). Monthly downloads continue over the lifespan of each guideline, but after six months may be influenced by more external factors, such as increased media attention on the topic of concussion after a high-profile athlete injury.

GItools considered in this study included the clinician summary, patient summary, case study, and slide set. Accesses were defined as the number of times the GItool file link was selected by users. Information on user identity and the number of unique users was not available. The AAN sports concussion guideline update was accompanied by additional GItools, in part because its recommendations were relevant to health professionals and educators outside of neurology [29]. Website usage statistics for each of its seven GItools from publication in March 2013 to January 2014 were collected, including the number of mobile application installations through the Apple and Google Play stores. Summary statistics were generated for GItool accesses including the number, frequency and 95% confidence interval (CI). The Chi-square test was used to investigate the significance of the difference in observed to expected number of accesses for each GItool assuming that accesses would be the same between the four tools. For the sports concussion guideline, Chi-square tests were used to compare pair-wise observed versus expected accesses for each of its seven GItools. *P* values <0.05 were considered statistically significant.

### Self-reported GItool use

The AAN randomly selected 800 of its 855 eligible members to complete an online survey (Additional file 1) regarding use of the sports concussion guideline and its related GItools six months after it was launched in October 2013. Eligible members included junior resident, junior fellow, associate, active, corresponding active, fellow, corresponding fellow, and honorary members who lived in the United States and were members of the Sports Neurology or Child Neurology Sections. Members were excluded if they were retired, still in medical school, had helped to develop the survey, or did not have a known email address. Participants were first notified of the survey via email on November 22, 2013. Follow-up reminders were sent to non-respondents on December 2, 2013. Data collection closed on December 9, 2013. Correlation between self-reported guideline implementation and GItool use was tested for significance using pairwise Chi-square comparisons.

## Results

### GItool accesses

**Fig. 1:**
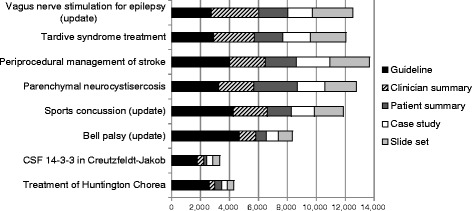
Absolute number of guideline and GItools accesses over six months for AAN guidelines published between July 2012 and August 2013

Figure 1 shows the absolute number of guideline and GItools accesses for the eight AAN guidelines published between July 2012 and August 2013, three of which were updates of existing guidelines while five were newly released guidelines [22–29]. On average, the clinician summary accounted for 29.0% (95% CI 28.7 to 29.4%) of accesses, the slide presentation accounted for 26.8% (95% CI 26.4 to 27.2%) of accesses, the patient summary accounted for 23.2% (95% CI 22.9 to 23.6%) and the case study accounted for 20.9% (95% CI 20.6 to 21.3%). The clinician summary was more frequently accessed than any of the other GItools (each *p* < 0.001); the slide set was more frequently accessed than the patient summary and clinical case example (both *p* < 0.001); and the patient summary was more frequently accessed than the clinical case example (*p* < 0.001).

**Fig. 2:**
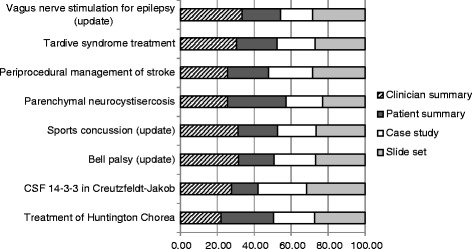
Proportion of accesses by type of GItool for each guideline

Figure 2 demonstrates differences in GItool accesses by guideline topic. For example, the patient summary was accessed more frequently than the physician summary for the Huntington Chorea [23] and parenchymal neurocystisercosis [25] guidelines. The proportion of accesses by type of GItool for each guideline ranged from 22.0 to 34.0% for the clinician summary, 14.0 to 32.0% for the patient summary, 17.0 to 26.0% for the case study, and 23.0 to 31.0% for the slide set.

**Fig. 3:**
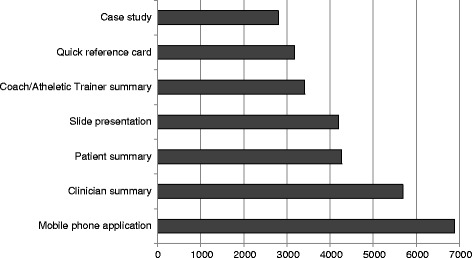
Absolute number of accesses for GItools offered with the sport concussion guideline from June 2013 to January 2014

Three additional GItools were created for the sports concussion guideline [29]: a coach/trainer summary, quick check reference card, and the Concussion Quick Check mobile phone application. Figure 3 shows that from June 2013 to January 2014, the most frequently accessed GItool for the sport concussion guideline was the mobile phone application, followed by the clinician summary, patient summary, and slide presentation. When considering the absolute number of accesses, all pair-wise comparisons were significant at a *p* < 0.001 level except for the patient summary and slide set (*p* = 0.48).

### Self-reported GItool use

Three members were removed from the survey sample due to invalid email addresses, for a final sample size of 797. The overall response rate was 21.8% (168/797). Responders were older (51.7 vs 49.7 years, *p* = 0.02) and more likely to be male (75.4% vs 67.6%, *p* = 0.05). Survey responders were also more likely to have fellow status (which means they were more senior).

Of survey respondents, 88.1% were aware of the sport concussion guideline prior to the survey and 78.6% said they had read the sport concussion guideline. With regards to GItools, over half reported having read the quick check reference card (51.2%). This was followed by the clinician summary (45.2%), patient summary (28.0%), mobile phone application (26.2%), coach/athletic trainer summary (20.2%), presentation slides (14.9%), and case study (9.5%).

Among respondents, 85.1% (143/168) answered the question “Have you implemented any of the guideline recommendations?” Of these, 83.2% (119/143) said they had implemented one or more recommendations. Of 24 individuals who had not implemented the recommendations, 75.0% (18/24) reported having no opportunity to do so. Table 1 shows that patterns of GItools accessed were similar between respondents who said they implemented the guideline compared with those who did not. Only presentation slides and the coach/athletic trainer summary were used significantly more frequently by guideline implementers compared with non-implementers.

**Table 1:** Differences in GItools access between those who said they had implemented and had not implemented the sport concussion guideline

GItool	Implemented (% of 119)	Not implemented (% of 24)	Chi-square *p*-value
Quick Check reference card	61.3	41.7	0.08
Clinician summary	54.6	33.3	0.06
Patient summary	34.5	16.7	0.09
Quick Check phone application	28.6	33.3	0.64
Coach/Athletic trainer summary	26.9	8.3	0.05
Presentation slides	20.2	4.2	0.03
Case study	12.6	4.2	0.19

## Discussion

This study was conducted to identify naturalistic access of GItools. Website use statistics from the first 6 months after publication and data from a self-report survey revealed that many types of GItools were accessed including clinician summaries, patient summaries, and presentation slides. The clinician summary was used significantly more than other types of GItools overall, although patterns of use varied by guideline topic. For the sports concussion guideline [29], website use statistics found that the mobile phone application was most frequently accessed (followed by the physician summary) while the self-report survey found that the reference card was the most frequently accessed GItool (also followed by the physician summary), perhaps reflecting the preferences of older physicians responding to the survey. Patterns of GItool access for the sport concussion guideline were similar between those who had and had not yet had an opportunity to implement the guideline, perhaps suggesting that physicians refer to GItools in preparation for future implementation. Alternatively, the lack of a difference between groups may reflect limited statistical precision due to small sample size (*n* = 24 for non-implementers). The proportion of GItool accesses was higher in implementers than non-implementers for all tools except the mobile phone app.

These naturalistic findings support results of interviews and focus groups exploring the preferences for use of GItools by health professionals. In focus groups and interviews with 62 medical directors about how to increase use of the American College of Occupational and Environmental Medicine’s guidelines, the need for quick reference tools was a high priority [30]. In interviews and focus groups with 20 family physicians about their preferences for guideline content and format, participants expressed the need for guideline summaries including charts, tables and algorithms [31]. Interviews with 28 health professionals from four intensive care units revealed that GItools such as checklists which could be quickly consulted as a reminder were viewed as enablers of guideline implementation [32]. In the Cochrane review referenced earlier, physician summaries of systematic reviews (print bulletins) resulted in greater adherence to evidence-based practice [13].

Our findings and those of others are aligned with known barriers to guideline use. A meta-review of 12 systematic reviews of factors that influence the implementation of guidelines found that guidelines which were easy to understand and apply were more likely to overcome individual physician barriers of insufficient time and lack of familiarity with guidelines [5]. A realist systematic review of 278 studies also found that guidelines were more “implementable” if they were available in multiple formats including summaries, algorithms, and graphics [33]. Cognitive science theory suggests that guidelines may be difficult to use because they present complex information that prescribes action which may not match contextual circumstances, individual knowledge and experience, and organizational capacity [34]. Easy-to-use summaries and other point-of-care GItools may therefore support various types of decisions including evidence-informed (based on featuring effectiveness data), experiential (based on eliciting professional judgment), and shared (resources that support shared decision-making with patients and caregivers) [34]. At the same time GItools may support various types of decision-making processes including intuitive (trigger or reconcile with previous experience) and analytic (create or simulate new mental models) decision-making [35].

Given these findings, the production of physician summaries should be a priority for guideline developers. While our study did not investigate preferences for summary format, guidance on the optimal content and format for evidence summaries is beginning to emerge. A series of research studies generated insight on the content and format of decision boxes, point-of-care tools that provide clinicians with research evidence about equivocal management options [36]. A systematic review of literature from medicine, psychology, design, and human factors engineering on the characteristics of guidelines that are associated with their use in practice generated three categories of recommendations for formatting guidelines or accompanying GItools: content should be vivid so that it stands out, intuitive so that it can be easily understood, and visual so that it can be quickly interpreted [37].

While the mobile phone application was the most accessed GItool in association with the sport concussion guideline according to website use statistics, only 26.2% of survey respondents said that they used the application. This may reflect the fact the survey respondents were older compared with non-responders, or the novelty of the application given that it was the AAN’s first guideline-based mobile phone application. Most research on the use of mobile phone applications has focused on their use by patients, for example, to support smoking cessation [38], or the self-management of chronic conditions such as asthma [39]. Therefore, further research is needed to examine the effectiveness of mobile applications as a mechanism for physician-based implementation of guidelines.

Patient summaries were accessed for all guidelines but showed the largest variation across guidelines. In general, research has established that physicians face many challenges in the practice of shared decision-making [40], however, the variability in access across guidelines suggests that other factors related to guideline topic or recommendations may be more relevant. For example, there were three guidelines for which the guideline itself was accessed more frequently than all the associated tools combined (Fig. 1). These three guidelines were both more focused (each including only a single question) and on conditions with a lower prevalence than those conditions covered in the other guidelines. While the association between GItool use and guideline topic and breadth must be confirmed through future research, these results may suggest that developers with limited resources should prioritize GItool development for highly prevalent conditions and/or more complex guidelines, suggestions that also have clear face validity. Case studies were the least utilized GItool across analyses, resulting in a decision at the AAN to discontinue production of this GItool.

Several issues limit the interpretation and application of these findings. GItools are freely available on the AAN website and website use statistics did not identify users. Thus, the type of user accessing the GItools is unknown and the number of times each GItool was accessed represents absolute rather than unique uses. While self-report survey data is subject to various types of bias, the website use statistics were largely corroborated by the survey on the use of the sport concussion guideline and its GItools. Furthermore, this was a naturalistic study based on website access and a survey and did not investigate physician preferences for GItools with interview or focus group techniques, thus providing limited insights into how to package preferred GItools. This limitation is also a strength, however, as it sheds light on the real-life access of GItools rather than just investigating the opinions of individuals invested enough in the topic to offer opinions as part of a formal research study. Caution should be taken in interpreting survey results as the response rate was only 21.8%. This level of response is typical for surveys but may overestimate familiarity with and use of the guideline and associated GItools if respondents familiar with the guideline were more likely to complete the survey. Finally, we examined GItool access over only the first 6 months after guideline publication. This time frame correlates with only the first stages of information diffusion – presentation to users – and does not represent the S-shaped cumulative adoptive curve demonstrating increasing adoption from early to late adopters over time [41]. It is possible that there could be meaningful differences in GItool use between early and late adopters which are not captured in this analysis.

The results of this study can help guideline developers, implementers and researchers understand the most commonly accessed GItools in practice, thus assisting these groups to optimize the development and impact of commonly used GItools, hopefully resulting in increased implementation down-stream. Ongoing research will build upon these findings by exploring the underlying reasons for GItool preferences, and how the content, format, use and impact of GItools can be improved. It is well recognized that a variety of interacting factors influence physician use of guidelines including the organizations and system within which they work [5, 6, 10, 11, 42]. However, if we are to attend to the many challenges that must be overcome to promote the use of guidelines, then use of guidelines by individual physicians at the point-of-care remains a priority. Thus these findings are of particular interest to guideline developers who often have no budget for dissemination and implementation, and must therefore prioritize which types of GItools to create as part of the cost of developing guidelines [7].

## Conclusions

Many types of GItools were accessed and used by physicians to support implementation of guidelines including clinician summaries, patient summaries, and presentation slides. Trends in GItool use were generally similar across analyses, though the sports concussion mobile phone app was accessed more frequently by website use statistics than by survey report, possibly reflecting the older age of survey respondents or interest due to novelty, rather than intended use. Overall accesses were fewer in focused guidelines addressing relatively less common conditions. Patterns of use were similar between physicians who had, and not yet had an opportunity to implement the guidelines. Clinician summaries were particularly highly accessed GItools across analyses, suggesting these should be a priority for guideline developers with limited resources. Patient summaries were more frequently accessed than physician summaries for some guidelines, however. The variation in GItool access according to guideline topic suggests the need for future research in understanding physician preferences overall and in relationship to guideline scope. Further research is also needed to investigate how to optimize GItool delivery via mobile app.

## Abbreviations

AAN: American academy of neurology
CI: Confidence interval
GItool: Guideline implementation tool
